# Differing natural killer cell, T cell and antibody profiles in antiretroviral-naive HIV-1 viraemic controllers with and without protective HLA alleles

**DOI:** 10.1371/journal.pone.0286507

**Published:** 2023-06-02

**Authors:** Ana Moyano, Bongiwe Ndlovu, Msizi Mbele, Kewreshini Naidoo, Nasreen Khan, Jaclyn K. Mann, Thumbi Ndung’u

**Affiliations:** 1 Africa Health Research Institute, KwaZulu-Natal, South Africa, Durban, South Africa; 2 HIV Pathogenesis Programme, The Doris Duke Medical Research Institute, School of Laboratory Medicine and Medical Sciences, University of KwaZulu-Natal, Durban, South Africa; 3 Division of Infection and Immunity, University College London, London, United Kingdom; 4 Ragon Institute of Massachusetts General Hospital, Massachusetts Institute of Technology and Harvard University, Cambridge, MA, United States of America; University of Verona, ITALY

## Abstract

Previous work suggests that HIV controllers with protective human leukocyte antigen class I alleles (VC+) possess a high breadth of Gag-specific CD8+ T cell responses, while controllers without protective alleles (VC-) have a different unknown mechanism of control. We aimed to gain further insight into potential mechanisms of control in VC+ and VC-. We studied 15 VC+, 12 VC- and 4 healthy uninfected individuals (UI). CD8+ T cell responses were measured by ELISpot. Flow cytometry was performed to analyse surface markers for activation, maturation, and exhaustion on natural killer (NK) cell and T cells, as well as cytokine secretion from stimulated NK cells. We measured plasma neutralization activity against a panel of 18 Env-pseudotyped viruses using the TZM-bl neutralization assay. We found no significant differences in the magnitude and breadth of CD8+ T cell responses between VC+ and VC-. However, NK cells from VC- had higher levels of activation markers (HLA-DR and CD38) (p = 0.03), and lower cytokine expression (MIP-1β and TNF-α) (p = 0.05 and p = 0.04, respectively) than NK cells from VC+. T cells from VC- had higher levels of activation (CD38 and HLA-DR co-expression) (p = 0.05), as well as a trend towards higher expression of the terminal differentiation marker CD57 (p = 0.09) when compared to VC+. There was no difference in overall neutralization breadth between VC+ and VC- groups, although there was a trend for higher neutralization potency in the VC- group (p = 0.09). Altogether, these results suggest that VC- have a more activated NK cell profile with lower cytokine expression, and a more terminally differentiated and activated T cell profile than VC+. VC- also showed a trend of more potent neutralizing antibody responses that may enhance viral clearance. Further studies are required to understand how these NK, T cell and antibody profiles may contribute to differing mechanisms of control in VC+ and VC-.

## Introduction

People living with HIV-1 have different rates of disease progression in the absence of antiretroviral therapy (ART), with a subset who rapidly progress to AIDS and a subset who is able to control infection. Multiple studies have linked HIV-1 control, or lack of it, to several virologic, immunologic and genetic factors [1–6]. Human leukocyte antigen (HLA) class I (HLA-I) is the most significant genetic determinant of clinical outcome in HIV-1 infection [7]. The expression of “protective” HLA-I alleles such as HLA-B*27, HLA-B*57, HLA-B*58:01, HLA-B*81:01 and HLA-A*74 is associated with low viral loads and slower progression to AIDS [7–15]. In contrast, the expression of “non-protective” HLA-I alleles such as HLA-B*08, HLA-B*58:02, and HLA-B*18, is associated with higher viral loads and rapid disease progression [8, 13]. HLA-I molecules present viral peptides to HIV-specific CD8+ cytotoxic T lymphocytes and the major mechanism by which individuals with protective HLA-I alleles control infection is thought to be through CD8+ T cell activity [1, 4, 16–18]. Furthermore, Gag-specific CD8+ T cell responses are associated with the relative control of viral replication [19–21]. However, escape mutations within Gag epitopes, or loss of breadth in Gag CD8+ T cell responses with or without associated escape mutations can lead to loss of viraemic control by individuals possessing these protective HLA-I alleles [22]. This indicates that control in individuals with protective HLA-I alleles is associated with the ability of their CD8+ T cells to control viral replication and loss of control may be precipitated by decreased breadth in Gag CD8+ T cell responses [22]. However, there are individuals without protective HLA-I alleles who maintain control and are characterised by CD8+ T cells with poor ability to suppress HIV-1 replication *ex-vivo*. The mechanisms of control independent of the CD8+ T cell response in these individuals are not yet fully understood [22].

Natural killer (NK) cells are a subset of peripheral lymphocytes that play a critical role in the innate immune response to virus-infected and tumour transformed cells [23]. NK cells can be subdivided into different subsets: CD56^dim^ (CD56+CD16+), CD56^bright^ (CD56+CD16-) and CD56^neg^ (CD56-CD16+) NK cells. CD56^dim^ (CD56+CD16+) are cytotoxic and produce perforin and granzyme B predominantly, while CD56^bright^ (CD56+CD16-) NK cells are immune-regulatory and secrete cytokines including IFN-γ, TNF-α, IL-10, and IL-13. CD56^neg^ (CD56-CD16+) NK cells are a dysfunctional subset [24, 25] and express low levels of the natural cytotoxicity receptors NKp30 and NKp46, have low expression of IFN-γ, and exhibit impaired cytotoxicity (based on the expression of Siglec-7) [26]. NK cells have multiple direct antiviral functions and also act as immune regulators [27, 28], through production of several cytokines and chemokines [29]. During HIV-1 infection there is a redistribution of NK cell subpopulations: in the acute phase there is an early depletion of the immune-regulatory subset (CD56^bright^), then ongoing viral replication is followed by a reduction of the cytotoxic subset (CD56^dim^) with a parallel increase in the dysfunctional subset (CD56^neg^) [24, 30].

Different studies have linked NK cell receptors and NK functionality with differences in HIV-1 disease progression [2, 5, 24, 25, 29, 31, 32]. Specific NK cell receptors (named killer immunoglobulin-like receptors or KIRs) in conjunction with protective HLA-I alleles have been shown to increase the likelihood of achieving controller status [24, 33–36]. Another study comparing controllers and long-term non-progressors (LTNP) evaluated the phenotypic and functional properties of NK cells, and suggested that LTNP could have a phenotypic and functional intermediate state between HIV-1 progressors and HIV-1 controllers [37]. In that study, IFN-γ expression was higher in the CD3-CD56+ NK subset in LTNP and controllers than in progressors and healthy donors, and this subset also had greater cytolytic activity in the non-progressor groups [37]. In addition, IFN-γ and chemokine production (MIP-α, MIP-β and RANTES) by NK cells has been associated with delayed disease progression [29]. While there is data linking specific NK receptors (in combination with specific HLA alleles), NK-mediated ADCC and NK-mediated production of cytokines and chemokines with HIV-1 control, it remains unknown if NK cell function differs between viraemic controllers (VC) with and without protective HLA-I alleles.

Previous studies have shown that only 10–30% of HIV-1 infected participants develop broadly neutralizing antibodies (bNAbs) that are capable of neutralizing viral variants from different HIV-1 subtypes [38–42]. Furthermore, the development of bNAbs is associated with the duration of infection, plasma viraemia, viral diversity, CD4+ T cell decline and the Fc effector functions [38–40, 42–44]. However, bNAbs do not appear to protect infected individuals from clinical disease progression [45]. Previous studies found that potent and broad neutralization activity was significantly higher in typical progressors compared to long-term non-progressors or elite controllers [40, 46–49]. However, higher neutralization breadth and potency has been observed among some viraemic non-progressors compared to typical progressors [46]. Viraemic non-progressors maintain moderate viraemia with a stable CD4+ T cell counts suggesting that the development of bNAbs could be enhanced by the continuous activation of B cells and the availability of sufficient CD4+ T helper cells that stimulate the formation of stable germinal center [46, 50]. Most bNAbs that develop in VC or long-term non progressors target the V3-glycan, although it is not clear whether they contribute to viral control [47, 51, 52].

A previous study reported on an HIV-1 subtype B VC that expressed protective HLA-I alleles including HLA-B*57:01 and HLA-B*27:05 and developed broad and potent neutralizing antibodies [52]. Three broadly neutralizing monoclonal antibodies (BG18, BG1 and NC37) were isolated from this participant and they targeted the N332-glycan supersite, the V2 apex and the CD4 binding site respectively. Most of the autologous viruses remained sensitive to neutralization by BG18 and NC37, however they escaped from BG1 suggesting a co-existence between sensitive strains and potent bNAbs [52]. These findings suggest that V3-glycan specific bNAbs may contribute to viral control in this individual. However, very little information is known about the role of broadly neutralizing antibodies in controllers with and without protective HLA alleles.

The goal of the present study was to gain further insight into potential mechanisms of control in VC with and without protective HLA-I alleles. We measured the breadth and magnitude of CD8+ T cell responses, frequencies of NK and T cell populations, surface markers for activation, maturation, and exhaustion profiling on these cellular populations, cytokine secretion from stimulated NK cells (as a measure of their function), and neutralization breadth and potency.

## Methodology

### Study participants

Patients were selected from several cohorts based in Durban including the Sinikithemba Cohort (E028/99) [11], the HIV Pathogenesis Programme (HPP) Acute Infection Cohort (E036/06) [53, 54] and the Females Rising through Education, Support and Health (FRESH) (BF131/11) [55], and the Elite Controllers Cohort (BE102/14). Participants were classified as VC if they maintained a viral load of <2,000 copies/ml for at least 12 months. We characterized 15 VC with protective HLA-I alleles (VC+) including HLA-B*27, HLA-B*57, HLA-B*58:01, HLA-B*81:01, and HLA-A*74 [7–9, 11, 13, 14, 22] and 12 VC without protective HLA-I alleles (VC-). A group of healthy uninfected individuals (UI) (n = 4) was included for the cellular phenotypic characterization. Study participants were ART-naïve. All samples selected for analysis were based on availability of PBMCs and plasma samples, and a minimum follow-up time of 12 months of viral control for VC. Participant characteristics are summarized in Table 1. Two participants from the VC+ group and one participant from VC- group were co-infected with *Mycobacterium tuberculosis* (MTB). However, the MTB co-infection information was only collected for the Sinikithemba cohort and information on other potential co-infections was not collected. All study subjects provided written informed consent and this study was approved by the University of KwaZulu-Natal’s Biomedical Research Ethics Committee.

**Table 1:** Study participant characteristics.

Parameter	Viraemic controllers with protective HLA-I alleles (VC+)	Viraemic controllers without protective HLA-I alleles (VC-)	P value
N	15	12	
Age (years at enrolment), Median (IQR)^a^	30 (20–69)	29.5 (20–51)	0.88
Females (n)^b^	10	12	0.05
Months of known control, Mean±SD^c^	42±25	39±20	0.71
Viral load (copies/ml), Median (IQR)^a^	65 (20–402)	126 (22–1102)	0.24
CD4 + T cell counts (cells/mm^3^), Median (IQR)^a^	623 (473–881)	582 (537–609)	0.39

^a^ Mann-Whitney test.

^b^ Fisher’s exact test.

^c^ T-test.

### HLA genotyping, CD4 T cell count and viral load testing

HLA-I genotyping as well as longitudinal viral load (VL) and CD4 T cell count data were available for all participants. Plasma viral loads were measured using either the Roche Amplicor Monitor assay (detection limit of 400 HIV-1 RNA copies/ml plasma) or the Roche Ultrasensitive assay (detection limit of 50 RNA copies/ml plasma), according to the manufacturer’s instructions. CD4 T cell counts were determined from fresh whole blood using Tru-Count technology and analyzed on a four-color flow cytometer (Becton Dickinson) according to the manufacturer’s instructions. DNA for HLA typing was extracted using a Puregene DNA isolation kit for blood (Gentra Systems) according to the manufacturer’s instructions. HLA-I genotyping was done by DNA PCR using sequence-specific primers as previously described [11].

### Antiretroviral drug measurements

To confirm that the participants were not taking antiretroviral drugs (ARVs) at the time points studied here, plasma samples were tested by the liquid chromatography coupled to tandem mass spectrometry (LC-MS/MS) as described previously [56]. The LC-MS/MS method is a highly sensitive technique for accurate quantification of ARVs in plasma [57, 58]. ARVs were not detected for any participant.

### Determination of the magnitude and breadth of CD8+ T cell responses

HIV-1 CD8+ T cell responses were enumerated from frozen whole peripheral blood mononuclear cells (PBMCs) by the gamma interferon (IFN-γ) enzyme-linked immunosorbent spot (ELISpot) assay as previously described [11, 59]. This assay method preferentially (although not exclusively) detects CD8+ T cell responses [11, 60]. Briefly, 100,000 PBMCs were stimulated with 410 consensus clade C 18-mer overlapping peptides (OLPs) covering the entire HIV-1 proteome using a matrix system of 11–12 peptides per pool. This was followed by a separate ELISpot assay using shorter optimal peptides to confirm which individual peptides were recognised within a reactive pool. The IFN-γ-secreting cells were counted using an ELISpot reader (AID-Diagnostika). The spot forming unit (SFU) value was multiplied by 10 (to express as per 1x10^6^ PBMC) and the background (the mean of the negative controls plus 3 times the standard deviation of the negative controls) was subtracted to calculate the SFU/well, which was then expressed as SFU per 1x10^6^ PBMC. Responses ≥100 SFU/1x10^6^ PBMC were considered to be positive.

### NK cell and T cell phenotypic and intracellular staining characterization

To perform cell phenotypic characterization, the surface markers used included those selecting for viable cells and specific cell populations (CD3 to select T cells, CD14 to exclude monocytes, CD19 to exclude B cells, and CD56 and CD16 to select NK cells), activation markers (CD69, CD38, HLA-DR and NKG2C), maturation markers (NKG2A and CD57), the exhaustion marker PD-1 and the natural cytotoxicity receptors NKp30, NKp44 and NKp46. Further to this, PBMCs were stimulated for analysis of NK cell degranulation (CD107a) and intracellular cytokine (IFN-γ, TNF-α and MIP-1β) expression was determined. Methods used were based on previously published protocols [61, 62].

Cryopreserved PBMCs were thawed, counted and resuspended at 1x10^6^ cells/ml in R10 media, and rested for 2 hours at 37°C, 5% CO_2_. After resting, cells were re-counted, centrifuged at 1800 rpm for 8 minutes and resuspended at 10x10^6^/ml in R10 media and 1x10^6^ cells were plated per well in a 96 well-plate. To phenotype the NK and T cells, the cells were centrifuged at 1800 rpm for 8 minutes, supernatant was discarded and cells were stained for 20 minutes at room temperature (RT) in the dark using LIVE/DEAD™ Fixable Aqua Dead Cell Stain Kit (Invitrogen) and specific monoclonal antibodies: CD3-BV785, CD14-BV650, CD19-BV650, CD56-Alexa Flour 700, CD16-APC-Cy7, CD69-PerCPCy5.5, CD38-BV711, HLA-DR-PE-CF594, NKG2C-PE, NKG2A-APC, CD57-FITC, PD-1-BV421, NKp30-PE-Cy7, NKp44-PE-Cy7, and NKp46-PE-Cy7 (S1 Table). Cells were then washed with PBS, fixed with Fix/Perm Medium A (Caltag), and resuspended in 200 μl PBS until acquisition.

To assess NK cell function, thawed PBMCs were rested for 6 hours at 37°C, 5% CO_2_ and were stimulated, followed by measurement of intracellular cytokines and the degranulation marker CD107a. The stimulation was performed by plating the PBMCs in R10 in a 96 well-plate at 1x10^6^ cells/well together with K562 cells, a tumour cell line, that lacks HLA expression (ATCC, Manassas, Virginia, USA; ATCC® CCL-243™), in a K562:PBMCratio of 1:10. The lack of HLA-I expression by K562 cells results in activation of NK cells since there is no HLA-I to provide an inhibitory signal to NK cells [63]. An unstimulated sample (PBMCs alone) was included as a baseline control. Brefeldin A (5 μg/ml, Sigma), Golgi stop (1:10 diluted in R10) (BD Biosciences) and CD107a-PE-Cy5 were added immediately to all wells (S1 Table). Cells were cultured for 18 hours at 37°C and 5% CO_2_. Following stimulation, PBMCs were washed with PBS and stained for 20 minutes at RT in the dark with LIVE/DEAD™ Fixable Aqua Dead Cell Stain and specific monoclonal antibodies: CD3-BV785, CD14-BV650, CD19-BV650, CD56-Alexa Flour 700 and CD16-APC-Cy7. Cells were washed with PBS, fixed with Fix/Perm Medium A for 20 minutes at RT in the dark. Cells were washed again, permeabilized (Fix/Perm B, Caltag) and stained for intracellular expression of IFN-γ-PECy7, TNF-α-BV605 and MIP-1β-PE (S1 Table) for 20 minutes at RT in the dark, washed and resuspended in PBS until acquisition.

Acquisition of cells was done on an LSR Fortessa flow cytometer (BD Biosciences). At least 200,000 events were acquired per sample when possible and analysed using FlowJo software (version 10.6.1). Compensation was calculated on the DIVA software using stained Anti-Mouse Ig, κ/Negative Control Compensation Particles (BD Biosciences). Fluorescence minus one (FMOs) were used to exclude background fluorescence in the gating strategies for each activation/differentiation marker. The gating strategies are shown in S1A Fig (cell subsets) and S1B Fig (cell surface markers and intracellular cytokines). Expression of differentiation/activation markers were compared between patient groups (VC+, VC- UI). NK cell expression of intracellular cytokines and the CD107a degranulation marker in response to stimulation was calculated by subtracting the unstimulated condition from the K562-stimulated condition, and also compared between patient groups.

### Neutralization assay

Neutralization activity was assessed in serum against a panel of 18 heterologous Env-pseudotyped viruses as previously described [39, 41]. Three-fold serial dilutions of heat-inactivated plasma were incubated with env-pseudotyped viruses for 1 hour at 37°C, 5% CO_2_. TZM-bl cells containing 5 mg/ml DEAE (Sigma-Aldrich) were added to all the wells and incubated for 48 hours at 37°C, 5% CO_2_. Culture media was removed from the wells and replaced with Bright-Glo Luciferase Assay System (Promega) and incubated in the dark for 2 minutes. The cell lysate was transferred into solid black 96-well plates and luminescence was measured in relative lights units using the Victor Nivo microplate reader (PerkinElmer). The neutralization antibody titers were reported as relative light units (RLUs) and the plasma dilutions needed to neutralize 50% of pseudoviruses. Neutralization breadth was calculated as the percentage of viruses neutralized with an inhibitory dilution (ID titer) > 50. Geometric mean titers (GMT) were evaluated by calculating the average serum dilution required to neutralize all the heterologous viruses tested. TZM-bl cells only were included as a negative control while the combination of each Env-pseudotyped virus and TZM-bl cells was included as a positive control.

### Data analysis

GraphPad Prism version 7 was used to construct graphs and perform statistical analyses. One-way ANOVA with Tukey post-hoc tests was used to assess if there were significant differences in breadth/magnitude of CD8+ T cell responses, cell populations, expression of surface markers or cytokine expression between the different patient groups. The Mann-Whitney test (unpaired, non-parametric t-test) was used to compare parameters when only 2 patient groups were compared. Pearson or Spearman tests were used for correlation analysis depending on whether or not the data was normally distributed. The difference in neutralization breadth, potency and antibody titers between VC+ and VC- was evaluated using the Mann-Whitney test. A significance cut off of p≤0.05 was used.

## Results

### No significant difference in CD8+ T cell response between VC+ and VC-

A previous study from our group reported that control in individuals with protective HLA-I alleles may be driven by Gag CD8+ T cell responses with potent viral inhibitory capacity, while control among individuals without protective alleles may be more durable and mediated by CD8+ T cell-independent mechanisms [22]. We therefore first sought to compare the breadth and magnitude of CD8+ T cell responses in an extended group of VCs (only 3 VC+ and 3 VC- study subjects in the current study were also in the previous study). We also investigated the activation and differentiation status of T cell (CD3+ cells) populations. To further explore potential CD8+ T cell-independent mechanisms of control, we compared the NK cell responses and antibody neutralization activity between VC- and VC+ groups.

**Fig 1:**
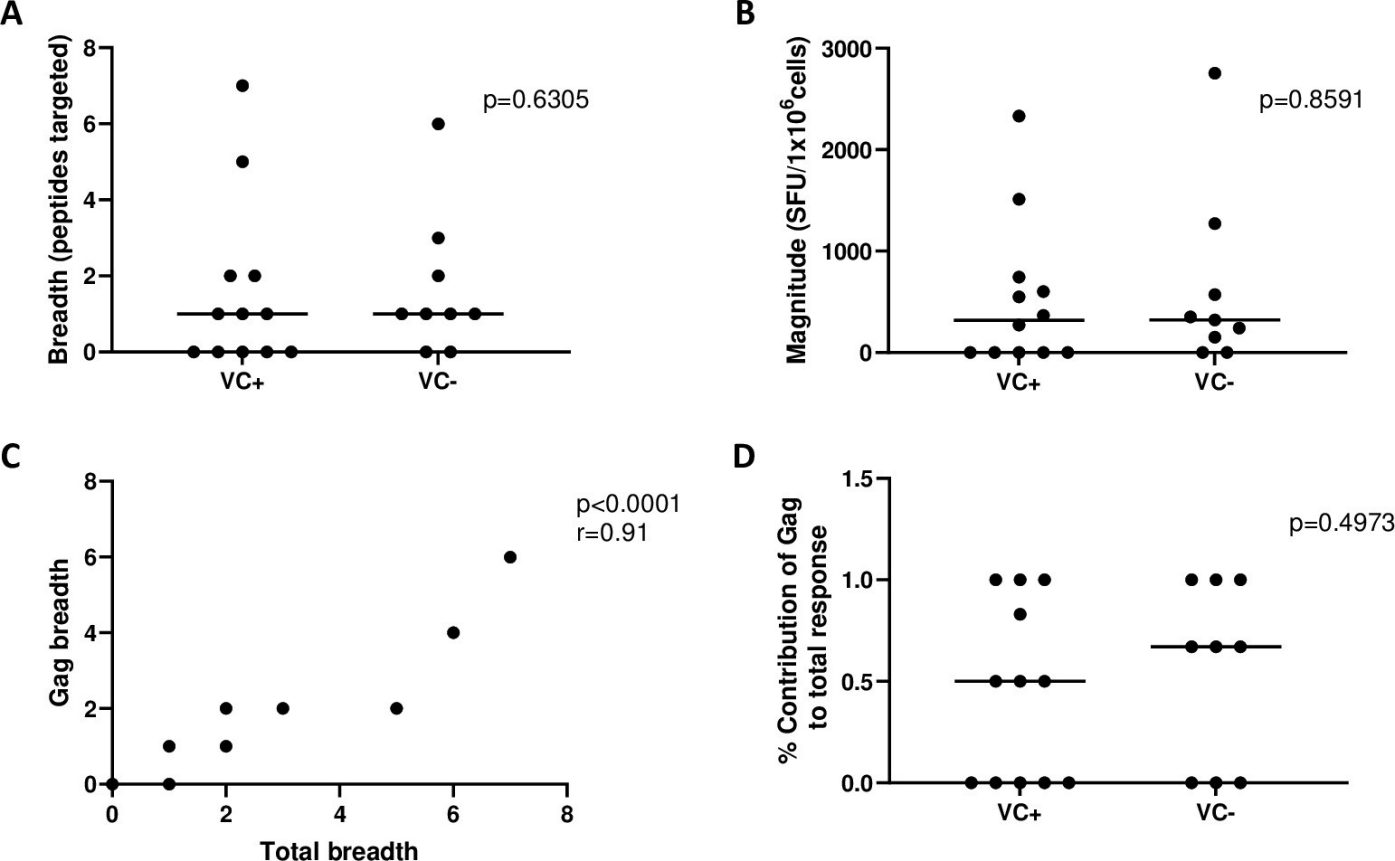
Analysis of HIV-specific CD8+ T cell (CD8+ T cell) responses. (A, B) Total breadth (A) and magnitude (B) of CD8+ T cell responses measured by the ELISpot assay were compared between viraemic controllers with protective alleles (VC+) and viraemic controllers without protective alleles (VC-) using the Mann-Whitney test. (C) Correlation of total breadth, Gag breadth as measured by the ELISpot assay for all groups is shown (Spearman’s correlation test). (D) Contribution of Gag CD8+ T cell responses to the total CD8+ T cell response as measured by the ELISpot assay was compared between VC+ and VC- using the Mann-Whitney test. The magnitude of CD8+ T cell responses to individual peptides was measured in spot forming units (SFU) per million cells. Bars represent the median.

There was no significant overall difference in total breadth and magnitude of CD8+ T cell responses between VC+ and VC- (p = 0.63 and p = 0.86, Mann-Whitney) (Fig 1A and 1B). Similar findings were obtained when the CD8+ T cell responses were analysed separately for each protein (Env, Gag, Nef, protease, reverse transcriptase) (S2 and S3 Tables).

Previous reports have shown a difference in the magnitude and breadth of Gag-specific CD8+ T cell responses between individuals with and without protective HLA-class I alleles when focussing only on epitopes in the most conserved region of Gag (amino acids 159–215, 261–300, 402–440) [64, 65]. Therefore, the analysis was narrowed down to overlapping peptides from those conserved regions only, however no significant difference in CD8+ T cell breadth and magnitude to the overlapping peptides in those regions was observed between the patient groups overall (Mann-Whitney, p = 0.88 and p = 0.96, respectively; S2 Fig). Interestingly, the majority of the CD8+ T cell responses in the VC were specific to Gag peptides and there was a strong correlation between total breadth of response and Gag-specific breadth (Spearman’s test, r = 0.91 and p<0.0001) (Fig 1C).

Gag-focussed CD8+ T cell responses as opposed to Env-focussed CD8+ T cell responses have been associated with lower viral loads [19], and CD8+ T cell responses to certain epitopes within Gag have previously been associated with slower disease progression [20]. Therefore, the contribution of the whole Gag CD8+ T cell response to the total CD8+ T cell response in an individual was calculated and compared between the patient groups. Here no significant difference was observed overall between VC+ and VC- (Mann-Whitney, p = 0.50) (Fig 1D). In summary, there was no difference in breadth or magnitude of CD8+ T cell responses as a whole or to specific viral proteins/regions. VC- has a slightly greater contribution of Gag CD8+ T cell responses to the total CD8+ T cell response than VC+, however this is not significant.

### NK cells from VC- have higher levels of activation markers than those from VC+ ex vivo

**Fig 2:**
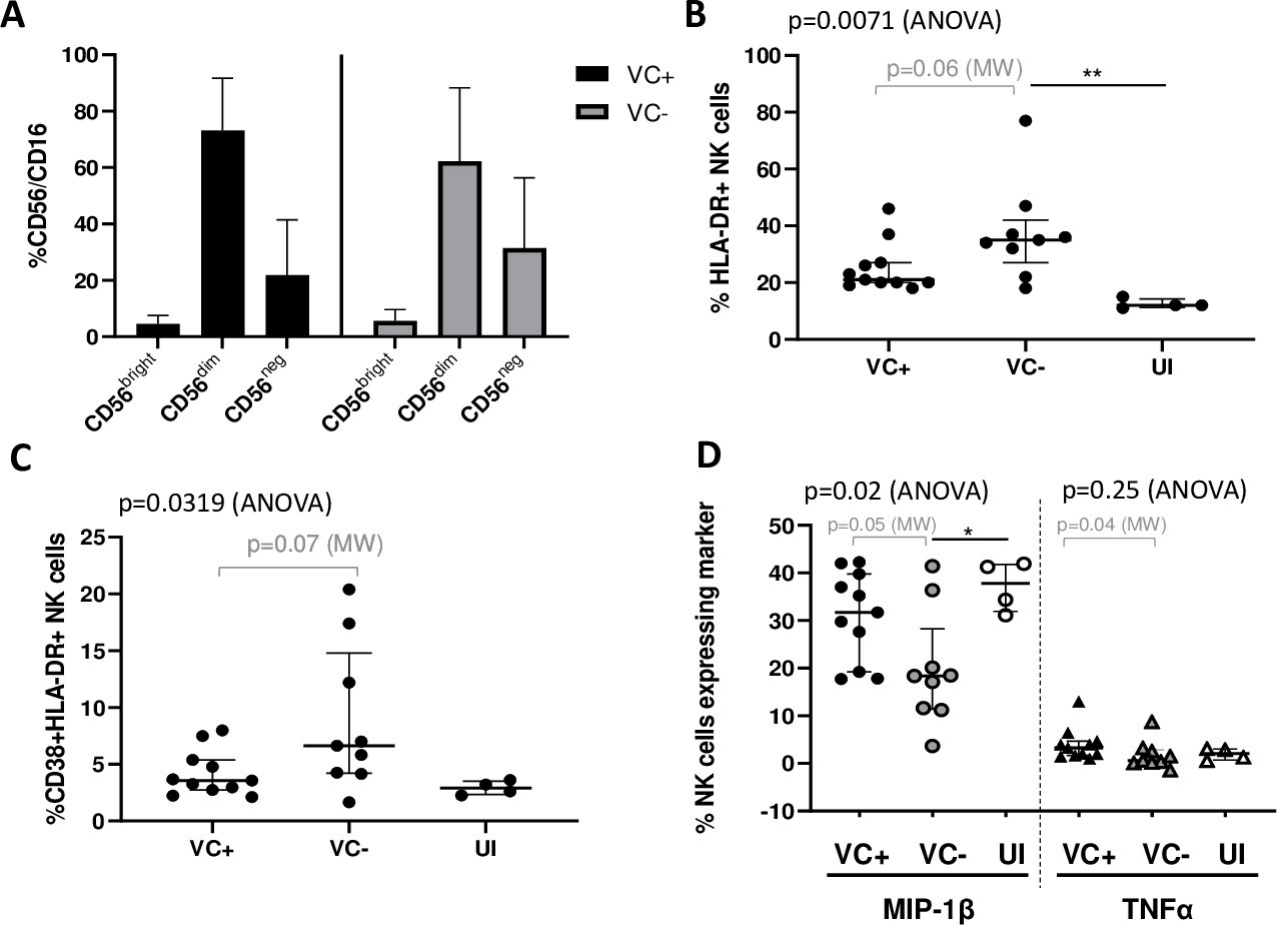
Flow cytometry analysis of NK cells. (A) Frequency of the different NK cell subpopulations, as measured by expression of CD56 and CD16 surface markers, was compared between viraemic controllers with protective alleles (VC+) and viraemic controllers without protective alleles (VC-). (B) Expression of the activation marker HLA-DR within the NK cell population was first compared between VC+ and VC-, and then between VC+, VC- and uninfected individuals (UI). Similarly, co-expression of CD38 and HLA-DR (C) and frequency of expression of the intracellular cytokines MIP-1β and TNF-α (D) within the NK cell population was compared between VC+, VC- and UI. The Mann-Whitney (MW) test (2 group comparisons) and one-way ANOVA (3 group comparisons) with the Tukey post-hoc test were used. Lines represent the median and bars show the interquartile range. ** represents p<0.01, * represents p<0.05 from the Tukey post-hoc test.

HIV-1 infection is characterized by the depletion of CD4+ T cells and the main immune driver of control of infection is CD8+ T cells [18]. However, recent studies suggest that NK cells might also play a significant role in control [24, 25, 30–32]. We characterized the phenotypes of NK cells using cell surface markers and measuring intracellular cytokines. We observed no significant differences in NK cell subset frequencies as distinguished by expression of CD56 and CD16 markers (CD56^bright^, CD56^dim^ and CD56^neg^) between VC+ and VC- (Mann-Whitney, p≥0.25; Fig 2A).

Similarly, there were no significant differences between VC+ and VC- in natural cytotoxicity receptors (NKp30, NKp44, and NKp46), maturation markers (NKG2A and CD57) or exhaustion markers (PD-1) (S4 Table). However, there was a tendency of higher expression of the activation marker HLA-DR in VC- compared to VC+ (p = 0.06) (Fig 2B). To provide further insight, we compared the expression of HLA-DR in VC+ and VC- to that in healthy uninfected controls (UI). Significant differences in HLA-DR expression were found between VC+, VC- and UI (ANOVA, p = 0.007), where VC- had higher HLA-DR expression than UI (p<0.01) (Fig 2B). This shows that, although there is a trend of higher activation in VC- than in VC+, both VC groups have somewhat higher activation than UI (only significant for VC-), which is to be expected given that the VC in this study are chronically infected individuals.

Since studies frequently measure HLA-DR co-expressed with CD38 as a marker of activated cells [66–69], we compared the frequency of CD38+HLA-DR+ NK cells between patient groups. Similar to HLA-DR+ cells, the co-expression of CD38 and HLA-DR was slightly higher in VC- compared to VC+ (Mann-Whitney, p = 0.07, Fig 2C). Furthermore, CD38 and HLA-DR co-expression was significantly different between VC+, VC- UI overall (ANOVA, p = 0.03) (Fig 2C). Although post-hoc tests were not significant, VC+ were more similar to UI, while VC- showed the highest CD38 and HLA-DR co-expression overall.

Taken together, these results suggest a trend for higher NK cell activation in VC- compared to VC+, whereas no differences in the frequency of the different NK cell subsets or other phenotypes were found between VC+ and VC-.

#### NK cells from VC+ express higher levels of intracellular cytokines in response to stimulation than those from VC-

To investigate the functionality of NK cells in response to stimulation, we incubated the PBMCs from VC with a tumour cell line and then measured NK cells for degranulation and intracellular cytokine expression. Degranulation marker (CD107a) and intracellular cytokines (IFN-γ, TNF-α and MIP-1β) in the unstimulated condition was subtracted from the stimulated condition. Although stimulation induced a slightly higher median CD107a expression in VC+ than VC- (19.9% vs 14.7% of NK cells), this was not statistically significant (Mann-Whitney, p = 0.23; S3A Fig). Similarly, there was no significant difference in IFN- γ expression between the VC groups (S3B Fig). However, VC+ expressed significantly higher MIP-1β and TNF-α (p = 0.05 and 0.04, Mann-Whitney, respectively) than VC- in response to stimulation (Fig 2D). To further interrogate the results showing a significant difference in expression of MIP-1β and TNF-α between the VC groups, we compared the expression of these cytokines in the VC groups to UI. VC+, VC- and UI have a significantly different expression of MIP-1β (ANOVA, p = 0.02). Both VC+ and VC- groups have lower median expression of MIP-1β compared to the UI group, although only VC- have significantly lower MIP-1β expression than UI (Fig 2D). However, TNF-α expression in both VC- and VC+ following stimulation was not significantly different from the UI group (ANOVA, p = 0.25) (Fig 2D). Overall, NK cells from VC+ show greater expression of MIP-1β and TNF-α upon stimulation than NK cells from VC-. Further, NK cells from VC+ were more similar to UI in terms of MIP-1β expression.

#### VC- T cells have a more activated and terminally differentiated profile than VC+ T cells

**Fig 3:**
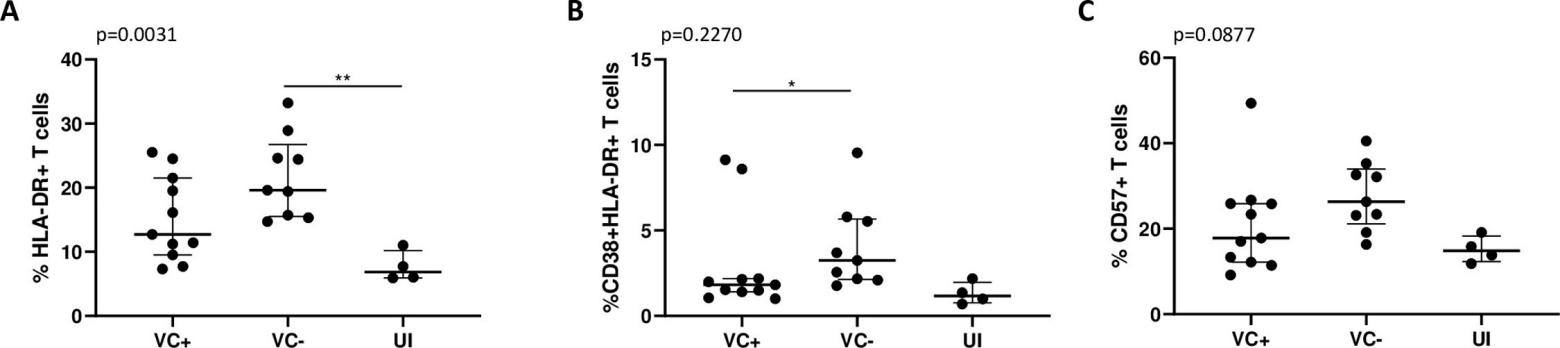
Flow cytometry analysis of T cells. (A) Expression of the activation marker HLA-DR within the T cell population is compared between viraemic controllers with protective alleles (VC+), viraemic controllers without protective alleles (VC-) and uninfected individuals (UI). Co-expression of CD38HLA-DR (B) and the senescence/terminal differentiation marker CD57 (C) within the T cell population is compared between VC+, VC- and UI. Mann-Whitney test and One-way ANOVA with Tukey post-hoc test were used. Lines represent the median and bars show the interquartile range. ** represents p<0.01, * represents p<0.05 from the Tukey post-hoc test.

Since T cells have been known to act as immune regulators and interact with NK cells, we measured the total frequency of CD3+ populations (T cells) and the expression of the activation markers CD69, CD38 and HLA-DR, as well as the terminal differentiation marker CD57 and the exhaustion marker PD-1, on T cells (S5 Table). There were no significant differences between VC+ and VC- for any of the markers. There was a tendency of higher expression of the activation marker HLA-DR (p = 0.07) in T cells from VC- when compared to VC+. This tendency was significant when compared to UI (ANOVA, p = 0.003), where VC had higher expression than UI and VC- had significantly higher HLA-DR expression than UI (p<0.01, Fig 3A). This shows that, similar to NK cells, although there is a trend of higher activation in VC- than in VC+, both VC groups have higher overall activation than UI, which is to be expected given that the VC in this study are chronically infected individuals.

Since HLA-DR co-expressed with CD38 in T cells is known to be a marker of immune activation [25, 66–68], we compared the frequency of CD38+HLA-DR+ T cells between patient groups. Similar to HLA-DR+ cells, there was significantly higher co-expression of CD38 and HLA-DR in VC- compared to VC+ (Mann-Whitney, p<0.05) (Fig 3B). However, VC+, VC- and UI did not differ significantly in CD38 and HLA-DR co-expression, although VC+ were more similar to UI and VC- showed the highest expression overall (ANOVA, p = 0.23) (Fig 3B).

In addition, we found a trend of higher expression of CD57 in VC- compared to VC+ (p = 0.09) (Fig 3C), were VC- have a higher proportion of CD57+ T cells. We compared the expression of CD57 in VC+, VC- and UI and, although VC+ were again more similar to UI and VC- had the highest CD57 expression, overall there was no significant difference in CD57 expression between our groups.

Altogether, these results suggest that VC- have HLA-DR expression, and CD38/HLA-DR co-expression, higher than VC+, who have values more similar to UI individuals, suggesting the CD3+ cells in VC- show a more activated profile when compared to UI and VC+ individuals. VC- also have a trend of higher CD57 expression than VC+ who again have more similar CD57 levels to UI individuals, suggesting the CD3+ population in VC- might show a higher terminally differentiated profile when compared to UI and VC+ individuals.

#### Neutralization breadth and potency in controllers with and without protective HLA- I alleles

**Fig 4:**
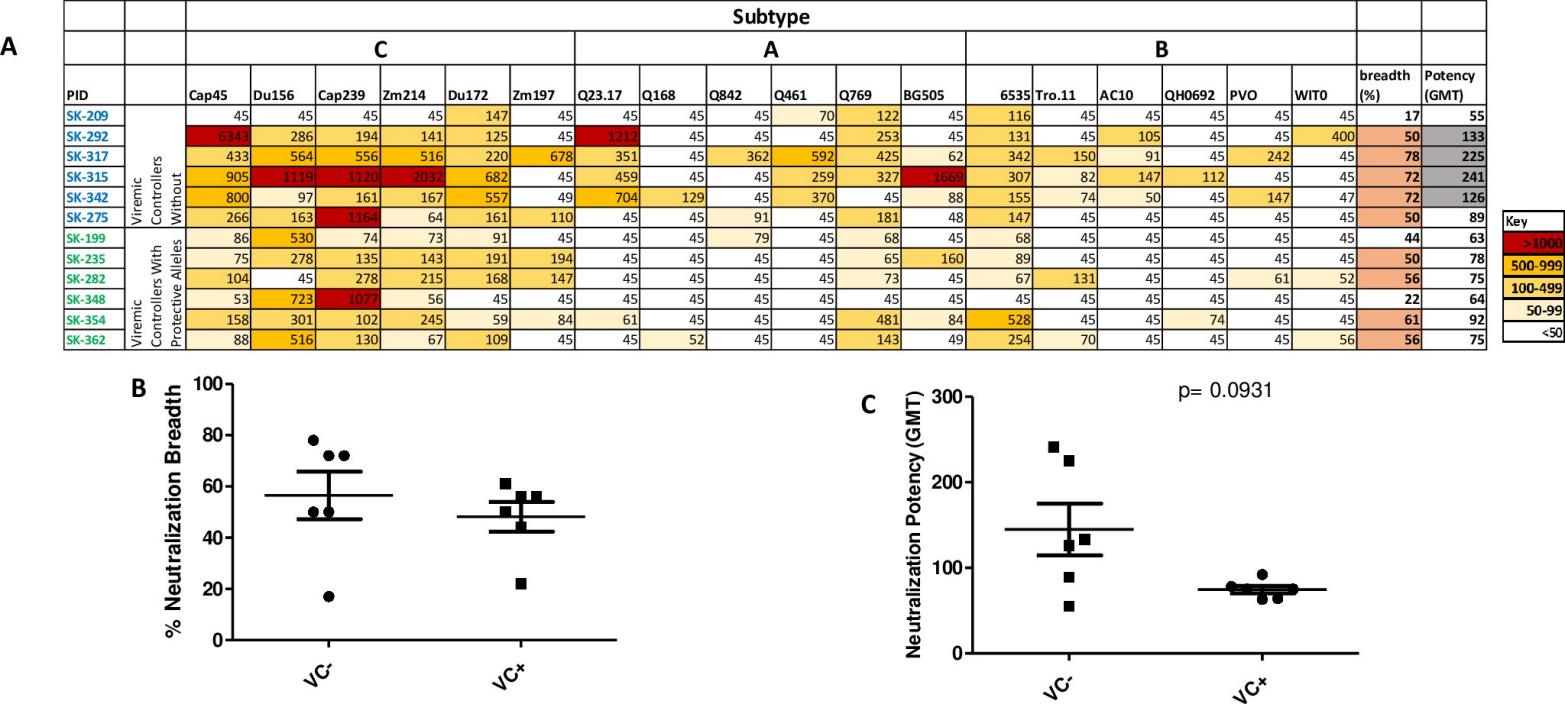
Neutralization profile of 12 viraemic controllers with and without protective HLA-class I alleles against a standard panel of 18 reference strains of subtypes A, B and C of different tiers (Tier 1, 2 and 3) at 3 years post enrollment (A). Comparison in neutralization breadth (B) and potency (C) between viraemic controllers with (VC+) and without (VC-) protective HLA-class I alleles. The highest virus titer (>1000) is shown in red and the lowest titer is shown in white (<50). Neutralization breadth greater or equal to 50% is shown in orange and neutralization potency greater or equal to 100 is shown in grey. The difference in neutralization breadth and potency between viraemic controllers with and without protective HLA alleles are shown in panel B and C respectively.

We next sought to determine whether there were differences in neutralization breadth and potency between VC+ and VC- groups. We screened 12 HIV-1 chronically infected VC for neutralization breadth and potency. A panel of 18 env-pseudotyped viruses was used to screen serum at 3 years post enrolment. Neutralization breadth was obtained when plasma neutralized at least 50% of the viruses tested. Overall, 9 of 12 (75%) VC showed broad neutralization activity, neutralizing more than 50% of the pseudoviruses tested. However, we found no difference in neutralization breadth between VC+ and VC- groups, although there was a trend for higher neutralization potency in the VC- group (p = 0.09) (Fig 4A). Overall, 5 out of 6 (83%) VC- developed neutralization breadth, neutralizing between 50–78% of heterologous viruses tested (Fig 4B), while only 4 out of 6 (67%) VC+ developed neutralization breadth and neutralized 50–61% of viruses (Fig 4B). Interestingly, 3 participants in the VC- group neutralized above 70% of viruses with a strong potency- (geometric mean titer (GMT)> 100); whereas participants in VC+ group only neutralized 50–60% of viruses with a weaker potency (GMT<100) (Fig 4C). Overall neutralization breadth was similar between the VC+ and VC- groups, although there was a trend for increased potency of neutralization in the VC- group.

## Discussion

In this study we examined the differences in immunological profiles between VC+ and VC- groups, by measuring CD8+ T cell breadth and magnitude, performing a phenotypic characterization of total NK cells and T cell populations, and measuring plasma HIV neutralization activity, to gain further insight into potential differences in mechanisms of control between VC+ and VC-.

We did not observe differences in the breadth or magnitude of CD8+ T cell responses measured by ELISpot between VC+ and VC-. The lack of significant differences between VC+ and VC- could be due to various reasons. Since both groups are matched for clinical parameters (VL, CD4 T cell counts, years of HIV-1 control), and the only difference is the presence of HLA-I protective alleles, we may expect the difference between both groups to be very subtle. Previous reports suggest that HIV-specific T cell responses cannot be adequately differentiated by ELISpot assays, and a follow-up with an *in vitro* HIV-1 suppression assay is recommended, as different studies have shown it is likely the most informative assay in the functional evaluation of CD8+ T cell responses [18, 70]. As reported by another study from our group, there was no difference in total breadth of CD8+ T cell responses or breadth of Gag CD8+ T cell responses as measured by ELISpot assay between VC+ and VC- but there was a significant difference in *ex vivo* virus inhibition capacity, where VC+ had CD8+ T cell responses with significantly more potent viral inhibitory capacity than VC-. In that study, viral suppression assays showed a clear difference in the mechanism of control between VC+ and VC- while the ELISpot analysis did not, supporting that HIV-1 suppression assays are preferable to assess HIV-specific CD8+ T cell responses especially when comparing groups with similar disease progression profiles [22].

We next explored the possibility that innate immune responses, and in particular NK cells, may differentiate the mechanisms of viral control between VC+ and VC-. When focusing on NK cells, our results showed no significant difference in the frequency of NK cell subsets, expression of natural cytotoxicity receptors (NCR), maturation markers or exhaustion markers between VC+ and VC-. We, however, observed a tendency of higher expression for the activation marker HLA-DR, and co-expression of CD38 and HLA-DR in NK cells from VC- when compared to VC+, suggesting that VC- have a more activated NK cell profile than VC+. CD38 has been described as a marker of disease progression, where the expression of CD38 on NK cells is associated with soluble and immunological factors found in advanced HIV-1 disease progression [25]. In our study, there was no significant difference in the expression of CD38 alone between VC+ and VC-, whereas we did observe higher expression of HLA-DR alone or in combination with CD38 in VC-, rendering it unclear based on the previous study [25] if this feature of VC- NK cells is unfavourable or not. Even though VC- had higher levels of activation markers, VC+ NK cells were more responsive upon stimulation than VC- in that they expressed higher levels of MIP-1β and TNF-α. Overall, the observation that VC- had increased levels of CD38+HLA-DR+ NK cells was unexpected and may require further approaches such as unbiased transcriptomic analyses of the cells to gain additional mechanistic insights. One possibility is that the higher HLA-DR in VC- NK cells could indicate that NK cells in VC- may be acting as more efficient antigen presenting cells (APCs) than those from VC+. Indeed, HLA-DR expressing NK cells have been shown to combine phenotypic characteristics of both NK cells and dendritic cells, and have been proposed to perform the function of professional APCs [71]. Further work is required to test the hypothesis that these cells may (at least in some circumstances) confer a beneficial antiviral effect as suggested by our data.

While we speculate that there may be differences in NK-mediated APC activity between VC+ and VC- based on the HLA-DR expression differences, our data does not indicate differences in cytolytic activity between the groups. We did not observe differences between NK cells from VC+ and VC- in expression of CD57, which has been associated with increasing prevalence of KIR+ and granzyme B+ cells [32], which suggests that the level of KIRs or granzyme B expressing cells is unlikely to be a distinguishing mechanism of control of the infection between the VC groups. Similarly, our results on CD107a expression, used as a functional marker for the identification of NK cell activity that correlates with TNF-α and IFN-γ secretion, and NK cell-mediated lysis of target cells [72], showed no difference between VC+ and VC-.

We further explored whether phenotypic characteristics of T cells might reveal additional potential different mechanisms of control between VC+ and VC-. Our results show a significant difference in T cell phenotype between VC+ and VC-, where VC- show a higher T cell activation (higher frequency of CD38/HLA-DR co-expression on T cells). Similar to NK cells, we observed a trend of higher expression of HLA-DR in VC- when compared to VC+, with both groups of VCs having significantly higher HLA-DR expression than UI. This suggests that, similar to NK cells, VC have a more activated T cell profile than UI and particularly more so in VC- than VC+. It is unexpected that VC- have a more activated profile than VC+ as previous studies have shown that CD38 and HLA-DR co-expression on CD8+ T cells is the strongest predictor for HIV-1 disease progression and higher expression of this profile is associated with adverse outcomes [25, 66–68], while, interestingly, in our previous study, we found that VC- were more likely to remain controllers on follow-up, compared to VC+ who were more likely to lose viraemic control [22]. Since HLA-DR expression correlates positively with the Ki-67 marker of cell cycling [73–75], we speculate that the trend of higher HLA-DR expression on T cells in VC- compared to VC+ might suggest that VC- can compensate for lower CD8+ T cell inhibitory capacity with a higher turnover of T cells. However, it is not clear if or how higher VC- T cell and NK cell activation can lead to a clinical benefit of VC- over VC+.

VC- also had slightly higher CD57 (terminal differentiation) expression on T cells than VC+, who were more similar to UI in terms of CD57 expression, suggesting that VC- have a slightly more terminally differentiated T cell profile. CD57 has been described as a marker of replicative senescence [76], and an increase of its expression on T cells and NK cells has been described as a general marker of proliferative inability and a history of more cell divisions. It is suggested that an increase in CD57+ T cells results from chronic antigen stimulation in HIV-1 infection [77, 78]. On the other hand, CD57 expression on CD4+ T cells identifies cytolytic cells, and its expression correlates with cytolytic granules, granzyme B and perforin expression [79]. The trend of differences observed in CD57 expression between VC+ and VC-, while not significant, might therefore indicate a slightly more cytolytic CD4+ T cell population in our VC-, however this cannot be definitely concluded, since the higher CD57 expression could be on CD8+ T cells and not CD4+ T cells. Of interest, CD8+ CD57+ T cells are associated with antibody neutralization breadth against HIV-1 in viraemic controllers, although the frequencies of CD8+CD57+, in that study, did not differ between VC+ and VC- [80].

Although CD57 expression was higher on T cells from VC-, and CD57 expression on CD8+ T cells has been associated with neutralisation breadth, we found no difference in neutralization breadth between VC+ and VC- groups. While there was no difference in neutralization breadth between VC- and VC+ groups, there was a trend of stronger neutralization potency in the VC- group. This is the first study to characterize the role of broad neutralization activity in HIV-1 control between individuals with and without protective HLA-class I alleles. Our findings suggest that neutralization breadth has very little impact in viral control. This is consistent with previous studies that reported that bNAbs are not associated with an improved clinical outcome [39, 40, 44, 45]. Although, a recent study found that a viraemic controller that expressed protective HLA-B*57:*01* and HLA-B*27:*05* had potent bNAbs that neutralized the circulating autologous viruses suggesting that they contributed to viral control [52].

Previous studies reported that potent and broad neutralization activity was significantly higher in typical progressors compared to LTNPs or elite controllers suggesting that the development of bNAbs may be driven by higher viral loads [40, 46–48]. Similarly, other studies have shown that plasma viral load and viral diversity may contribute to the development of bNAbs [40, 42, 45]. Nevertheless, it is interesting that bNAbs were detected in elite controllers [52] and LTNPs [40, 46–48] suggesting that other factors may also be important in the development of bNAbs. While there was no statistically difference in neutralisation breadth between VC- and VC+ groups observed in the present study, there was a trend of stronger neutralization potency in the VC- group and there were some VC- individuals with both high neutralization breadth (> 70%) and potency (GMT > 100) while this was not the case in the VC+ group. This suggests that neutralization activity may contribute to viral control in at least some VC-. It is also possible that serum antibodies may have augmented other effector functions including the antibody dependent cellular cytotoxicity (ADCC) or antibody-dependent cellular phagocytosis (ADCP). Further studies with a larger sample size that evaluate epitope-specific antibodies and other antibody-mediated effector functions are required to confirm these findings.

Some limitations of our study should be noted. Firstly, our reduced sample size makes interpretation of the data difficult and we should be cautious with drawing definite conclusions. In addition, we were not able to perform analysis of immune cell subset frequencies for individual participants and such differences could potentially result in interactions that alter phenotypic markers on immune cells, instead of the differences being intrinsic to cells in vivo. Another limitation is that we did not include flow cytometry markers to differentiate between CD4+ and CD8+ T cells, as a result of prioritizing our analysis on potential differences within the NK cell population. Thus, our findings apply to the whole T cell population and further analysis is needed to distinguish between the CD4+ and CD8+ subsets.

## Conclusions

While CD8+ T cell response did not distinguish VC+ from VC-, we observed that VC- have a more activated NK cell profile with lower cytokine expression, and a more terminally differentiated and activated T cell profile than VC+, and that VC- showed a trend of more potent neutralizing antibody responses that may enhance viral clearance. A possible explanation for our results is that the increased CD38+HLA-DR+ NK cells in VC- may represent NK cells more efficient as APCs, which may then imply better antiviral activity as a consequence of interaction with activated and terminally differentiated population of T cells observed in VC-. Further studies are required to understand how these NK, T cell and antibody profiles may contribute to differing mechanisms of control in VC+ and VC-. Considering that we observed heightened activation of both NK cells and T cells in VC-, it is possible that there is overall higher immune activation in VC- when compared to VC+, and it is likely important to address the inflammatory status in further studies. In addition, we believe that further studies including transcriptional profiling and immune cell type profiling, as well as including different patient groups (progressors virally suppressed on ART) for comparison, could aid in understanding the differences between VC- and VC+ observed in this study.

## Supporting information

S1 Fig Gating strategy for NK cell subsets and T cells. Determination of the proportion of lymphocytes (a), single cells (b), viable cells (c), CD3+CD14-CD19- cells (T cells) (d), CD3-CD14-CD19- cells (e), total NK cells (f), CD56^bright^ (g), CD56^dim^ (h) and CD56^neg^ (i). (B) Gating strategy for cell surface markers in NK cells and T cells and intracellular cytokine staining (ICS) in NK cells. Flow cytometry representation of the gating strategy for surface markers (top panel), co-expression of CD38 and HLA-DR (middle panels) and ICS (bottom panels). CD38* is used as a representation, and the same strategy was used for CD69, HLA-DR, NKG2C, NKG2A, CD57, PD-1, NKp30, NKp44 and NKp46. The quadrants for co-expression were drawn using the Fluorescence minus one (FMO) sample. For ICS the gates on both unstimulated and stimulated condition were done and then values were calculated by subtracting the unstimulated condition background; MIP-1β** is used as a representation, and the same strategy was used for IFN-γ, TNF-α and CD107a. Gate “a” represents the CD3+ population (T cells), gate “b” represents CD3- population and gate “c” represents the NK cell population.
(TIF)

S2 Fig Total breadth and magnitude of CD8+ T cell responses to epitopes in the most conserved region of Gag. Total breadth (A) and magnitude (B) of CD8+ T cell responses focussing only on epitopes in the most conserved region of Gag (amino acids 1–56, 57–96 and 97–135), measured by the ELISpot assay were compared between viraemic controllers with protective alleles (VC+), viraemic controllers without protective alleles (VC-) using the Mann-Whitney test. The magnitude of CD8+ T cell responses to individual peptides was measured in spot forming units (SFU) per million cells. Bars represent the median.
(TIF)

S3 Fig Flow cytometry analysis of the expression of the degranulation marker CD107a and intracellular cytokine IFN-gamma (IFN-γ) in the NK cell population. Frequency of expression of the CD107a (A) and IFN-γ (B) in the NK cell population was compared between viraemic controllers with protective alleles (VC+), viraemic controllers without protective alleles (VC-) and uninfected individuals (UI). ANOVA was used to compare expression between the 3 groups while the Mann-Whitney (MW) test was used to compare expression between VC+ and VC-. Lines and bars represent the median and interquartile range, respectively.
(TIF)

S1 Table Antibodies used for cell phenotypic and intracellular staining characterization. (DOCX)

S2 Table Breadth of total HIV-specific CD8+ T cell responses and by individual HIV proteins. (DOCX)

S3 Table Magnitude of total HIV-specific CD8+ T cell responses and by individual proteins. (DOCX)

S4 Table Percentage of expression of different surface markers and intracellular cytokines within the total NK cell population. NK cell expression of intracellular cytokines and the CD107a degranulation marker in response to stimulation was calculated by subtracting the unstimulated condition from the K562-stimulated condition, and also compared between patient groups.
(DOCX)

S5 Table Percentage of expression of different surface markers and intracellular cytokines within the CD3+ population. (DOCX)
