# Supplementary figures and images for: Differing natural killer cell, T cell and antibody profiles in antiretroviral-naive HIV-1 viraemic controllers with and without protective HLA alleles
Source: PLoS One. 2023 Jun 2;18(6):e0286507. doi: 10.1371/journal.pone.0286507 (PMC10237385; doi:10.1371/journal.pone.0286507)

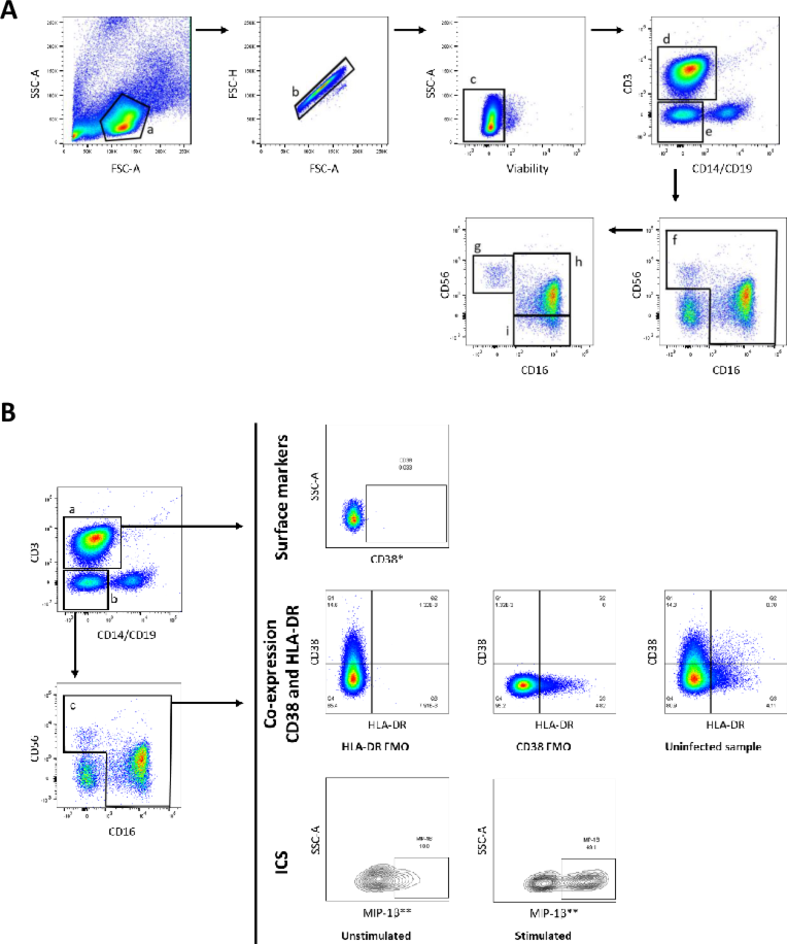

Supplement: S1 Fig — Determination of the proportion of lymphocytes (a), single cells (b), viable cells (c), CD3+CD14-CD19- cells (T cells) (d), CD3-CD14-CD19- cells (e), total NK cells (f), CD56bright (g), CD56dim (h) and CD56neg (i). (B) Gating strategy for cell surface markers in NK cells and T cells and intracellular cytokine staining (ICS) in NK cells. Flow cytometry representation of the gating strategy for surface markers (top panel), co-expression of CD38 and HLA-DR (middle panels) and ICS (bottom panels). CD38* is used as a representation, and the same strategy was used for CD69, HLA-DR, NKG2C, NKG2A, CD57, PD-1, NKp30, NKp44 and NKp46. The quadrants for co-expression were drawn using the Fluorescence minus one (FMO) sample. For ICS the gates on both unstimulated and stimulated condition were done and then values were calculated by subtracting the unstimulated condition background; MIP-1β** is used as a representation, and the same strategy was used for IFN-γ, TNF-α and CD107a. Gate “a” represents the CD3+ population (T cells), gate “b” represents CD3- population and gate “c” represents the NK cell population. (TIF) [file pone.0286507.s001.tif]

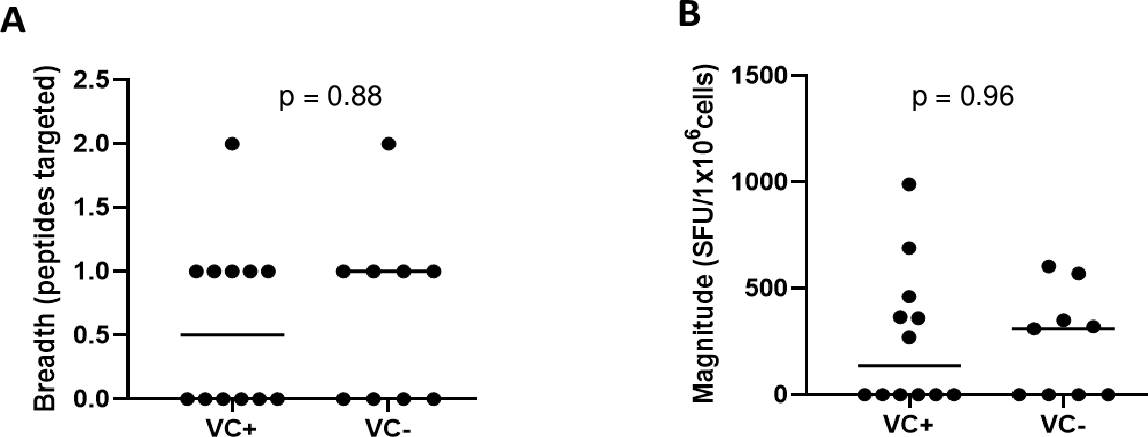

Supplement: S2 Fig — Total breadth (A) and magnitude (B) of CD8+ T cell responses focussing only on epitopes in the most conserved region of Gag (amino acids 1–56, 57–96 and 97–135), measured by the ELISpot assay were compared between viraemic controllers with protective alleles (VC+), viraemic controllers without protective alleles (VC-) using the Mann-Whitney test. The magnitude of CD8+ T cell responses to individual peptides was measured in spot forming units (SFU) per million cells. Bars represent the median. (TIF) [file pone.0286507.s002.tif]

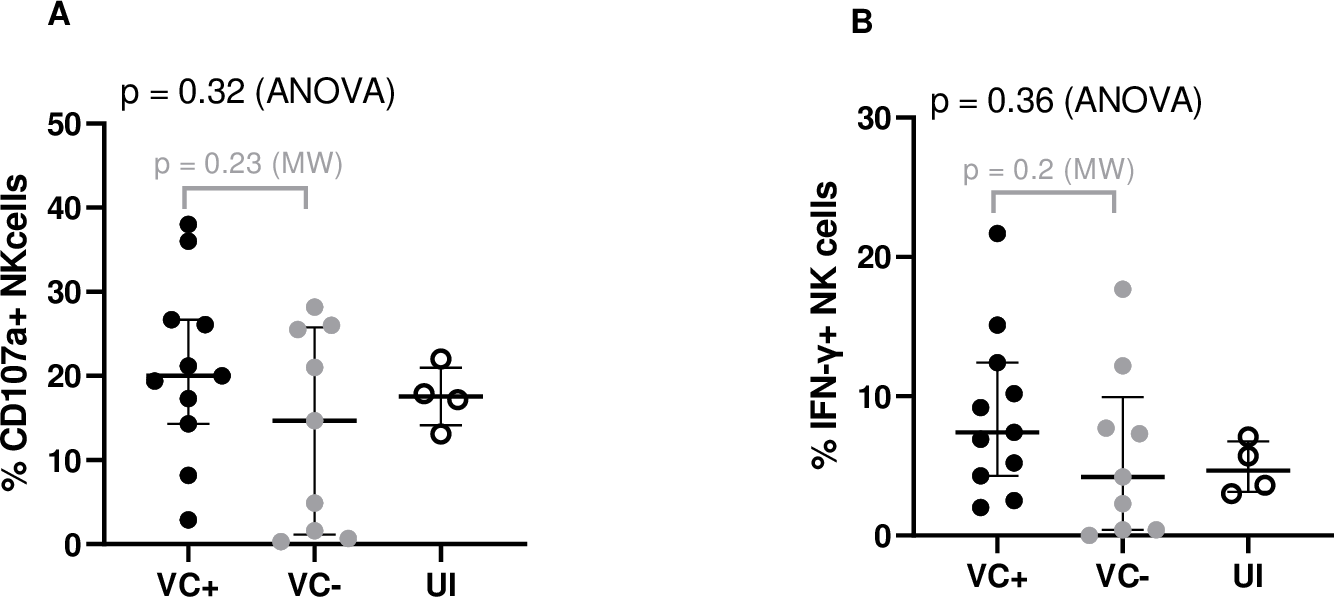

Supplement: S3 Fig — Frequency of expression of the CD107a (A) and IFN-γ (B) in the NK cell population was compared between viraemic controllers with protective alleles (VC+), viraemic controllers without protective alleles (VC-) and uninfected individuals (UI). ANOVA was used to compare expression between the 3 groups while the Mann-Whitney (MW) test was used to compare expression between VC+ and VC-. Lines and bars represent the median and interquartile range, respectively. (TIF) [file pone.0286507.s003.tif]
