# Supplementary material for: Differing natural killer cell, T cell and antibody profiles in antiretroviral-naive HIV-1 viraemic controllers with and without protective HLA alleles
Source: PLoS One. 2023 Jun 2;18(6):e0286507. doi: 10.1371/journal.pone.0286507 (PMC10237385; doi:10.1371/journal.pone.0286507)
Supplement: S1 Table — (DOCX) [file pone.0286507.s004.docx]

**S1 Table:** **Antibodies used for cell phenotypic and intracellular staining characterization.**

| Markers | Fluorochrome | Manufacturer | Clone | Dilution |
| --- | --- | --- | --- | --- |
| CD3 | Brilliant Violet (BV)785 | Biolegend | OKT3 | 1:20 |
| CD14 | BV 650 | Biolegend | M5E2 | 1:20 |
| CD19 | BV 650 | Biolegend | HIB19 | 1:20 |
| CD56 | Alexa Fluor 700 | BD Biosciences | B159 | 1:25 |
| CD16 | Allophycocyanin (APC)/Cy7 | BD Biosciences | 3G8 | 1:40 |
| CD69 | Peridinin Chlorophyll Protein (PerCP)/Cy5.5 | Biolegend | FN50 | 1:20 |
| CD57 | Fluorescein isothiocyanate (FITC) | BD Biosciences | NK-1 | 1:10 |
| PD-1 | BV 421 | Biolegend | EH12.2H7 | 1:20 |
| NKG2A | APC | Beckman Coulter | Z199 | 1:10 |
| CD38 | BV 711 | Biolegend | HIT2 | 1:20 |
| HLA-DR | Phycoerythrin (PE)/CF594 | BD Biosciences | G46-6 | 1:50 |
| NKG2C | PE | R&D Systems | 134591 | 1:10 |
| NKp30 | PE/Cy7 | Biolegend | P30-15 | 1:10 |
| NKp44 | PE/Cy7 | Biolegend | P44-8 | 1:10 |
| NKp46 | PE/Cy7 | Biolegend | 9E2 | 1:10 |
| CD107a | PE/Cy5 | BD Biosciences | H4A3 | 1:60 |
| TNF-α | BV 605 | Biolegend | MAb11 | 1:20 |
| MIP-1β | PE | BD Biosciences | D21-1351 | 1:20 |
| IFN-γ | PE/Cy7 | Biolegend | B27 | 1:20 |
