# Supplementary material for: Differing natural killer cell, T cell and antibody profiles in antiretroviral-naive HIV-1 viraemic controllers with and without protective HLA alleles
Source: PLoS One. 2023 Jun 2;18(6):e0286507. doi: 10.1371/journal.pone.0286507 (PMC10237385; doi:10.1371/journal.pone.0286507)
Supplement: S2 Table — (DOCX) [file pone.0286507.s005.docx]

**S2 Table: Breadth of total HIV-specific CD8+ T cell responses and by individual HIV proteins.**

|  |  | Number of peptides targeted | | | | | | | | | | | | |
| --- | --- | --- | --- | --- | --- | --- | --- | --- | --- | --- | --- | --- | --- | --- |
| Role^a^ | **Patient identifier** | **Total** | **Gag** | | **p24** | | **Nef** | | **Protease** | | **RT** | | **Env** | |
| VC+ | SK-235 | 1 | | 1 | | 1 | | 0 | | 0 | | 0 | | 0 |
| VC+ | SK-362 | 0 | | 0 | | 0 | | 0 | | 0 | | 0 | | 0 |
| VC+ | SK-354 | 0 | | 0 | | 0 | | 0 | | 0 | | 0 | | 0 |
| VC+ | SK-282/206-30-0020-0 | 1 | | 0 | | 0 | | 1 | | 0 | | 0 | | 0 |
| VC+ | 111-30-0005-0 | 1 | | 1 | | 1 | | 0 | | 0 | | 0 | | 0 |
| VC+ | 111-30-0015-0 | 0 | | 0 | | 0 | | 0 | | 0 | | 0 | | 0 |
| VC+ | SK-469/206-30-0011-0 | 2 | | 1 | | 1 | | 0 | | 1 | | 0 | | 0 |
| VC+ | AS-30-0018 | 7 | | 6 | | 4 | | 1 | | 0 | | 0 | | 0 |
| VC+ | SK-481/206-30-0007-0 | 2 | | 1 | | 1 | | 0 | | 0 | | 1 | | 0 |
| VC+ | SK-490/206-30-0012-0 | 0 | | 0 | | 0 | | 0 | | 0 | | 0 | | 0 |
| VC+ | SK-453 | 0 | | 0 | | 0 | | 0 | | 0 | | 0 | | 0 |
| VC+ | FRESH 127-33-0397-268 | 5 | | 2 | | 2 | | 0 | | 0 | | 3 | | 0 |
| VC- | SK-209 | 2 | | 2 | | 2 | | 0 | | 0 | | 0 | | 0 |
| VC- | SK-275 | 0 | | 0 | | 0 | | 0 | | 0 | | 0 | | 0 |
| VC- | SK-317 | 1 | | 1 | | 1 | | 0 | | 0 | | 0 | | 0 |
| VC- | 111-30-0041-0 | 1 | | 0 | | 0 | | 1 | | 0 | | 0 | | 0 |
| VC- | SK-452/206-30-0004-0 | 6 | | 4 | | 2 | | 1 | | 0 | | 0 | | 1 |
| VC- | SK-470/206-30-0005-0 | 1 | | 1 | | 1 | | 0 | | 0 | | 0 | | 0 |
| VC- | 206-30-0024 | 0 | | 0 | | 0 | | 0 | | 0 | | 0 | | 0 |
| VC- | SK-475/206-30-0002-0 | 1 | | 1 | | 1 | | 0 | | 0 | | 0 | | 0 |
| VC- | FRESH 127-33-0035-039 | 3 | | 2 | | 2 | | 0 | | 0 | | 1 | | 0 |

^a^ VC+, Viraemic controller with protective HLA-I alleles; VC-, Viraemic controllers without protective HLA-I alleles.
