# Supplementary material for: Differing natural killer cell, T cell and antibody profiles in antiretroviral-naive HIV-1 viraemic controllers with and without protective HLA alleles
Source: PLoS One. 2023 Jun 2;18(6):e0286507. doi: 10.1371/journal.pone.0286507 (PMC10237385; doi:10.1371/journal.pone.0286507)
Supplement: S3 Table — (DOCX) [file pone.0286507.s006.docx]

**S3 Table: Magnitude of total HIV-specific CD8+ T cell responses and by individual proteins.**

|  |  | Magnitude (spot forming units/million cells) | | | | | | |  |
| --- | --- | --- | --- | --- | --- | --- | --- | --- | --- |
| Role | **Patient identifier** | **Total** | **Gag** | **p24** | **Nef** | **Protease** | **RT** | **Env** | |
| VC+ | SK-235 | 365 | 365 | 365 | 0 | 0 | 0 | 0 | |
| VC+ | SK-362 | 0 | 0 | 0 | 0 | 0 | 0 | 0 | |
| VC+ | SK-354 | 0 | 0 | 0 | 0 | 0 | 0 | 0 | |
| VC+ | SK-282/206-30-0020-0 | 600 | 0 | 0 | 600 | 0 | 0 | 0 | |
| VC+ | 111-30-0005-0 | 270 | 270 | 270 | 0 | 0 | 0 | 0 | |
| VC+ | 111-30-0015-0 | 0 | 0 | 0 | 0 | 0 | 0 | 0 | |
| VC+ | SK-469/206-30-0011-0 | 743 | 462 | 462 | 0 | 281 | 0 | 0 | |
| VC+ | AS-30-0018 | 2330 | 2020 | 1720 | 310 | 0 | 0 | 0 | |
| VC+ | SK-481/206-30-0007-0 | 549 | 361 | 361 | 0 | 0 | 188 | 0 | |
| VC+ | SK-490/206-30-0012-0 | 0 | 0 | 0 | 0 | 0 | 0 | 0 | |
| VC+ | SK-453 | 0 | 0 | 0 | 0 | 0 | 0 | 0 | |
| VC+ | FRESH 127-33-0397-268 | 1510 | 820 | 820 | 0 | 0 | 690 | 0 | |
| VC- | SK-209 | 570 | 570 | 570 | 0 | 0 | 0 | 0 | |
| VC- | SK-275 | 0 | 0 | 0 | 0 | 0 | 0 | 0 | |
| VC- | SK-317 | 350 | 350 | 350 | 0 | 0 | 0 | 0 | |
| VC- | 111-30-0041-0 | 150 | 0 | 0 | 150 | 0 | 0 | 0 | |
| VC- | SK-452/206-30-0004-0 | 2753 | 1544 | 864 | 518 | 0 | 0 | 691 | |
| VC- | SK-470/206-30-0005-0 | 320 | 320 | 320 | 0 | 0 | 0 | 0 | |
| VC- | 206-30-0024 | 0 | 0 | 0 | 0 | 0 | 0 | 0 | |
| VC- | SK-475/206-30-0002-0 | 242 | 242 | 242 | 0 | 0 | 0 | 0 | |
| VC- | FRESH 127-33-0035-039 | 1272 | 938 | 938 | 0 | 0 | 334 | 0 | |

^a^ VC+, Viraemic controller with protective HLA-I alleles; VC-, Viraemic controllers without protective HLA-I alleles.
