# Supplementary material for: Differing natural killer cell, T cell and antibody profiles in antiretroviral-naive HIV-1 viraemic controllers with and without protective HLA alleles
Source: PLoS One. 2023 Jun 2;18(6):e0286507. doi: 10.1371/journal.pone.0286507 (PMC10237385; doi:10.1371/journal.pone.0286507)
Supplement: S4 Table — NK cell expression of intracellular cytokines and the CD107a degranulation marker in response to stimulation was calculated by subtracting the unstimulated condition from the K562-stimulated condition, and also compared between patient groups. (DOCX) [file pone.0286507.s007.docx]

**S4 Table: Percentage of expression of different surface markers and intracellular cytokines within the total NK cell population.** NK cell expression of intracellular cytokines and the CD107a degranulation marker in response to stimulation was calculated by subtracting the unstimulated condition from the K562-stimulated condition, and also compared between patient groups.

|  | |  | |  | | % expression | | | | | | | | | | | |
| --- | --- | --- | --- | --- | --- | --- | --- | --- | --- | --- | --- | --- | --- | --- | --- | --- | --- |
| Patient identifier | **Sex** | | **Role^a^** | | **CD38+** | **HLA-DR+** | **CD57+** | **CD69+** | **HLA-DR+ CD38+** | **NKG2A+** | **NKG2C+** | **NKp30 /p44/p46+** | **PD-1+** | **CD107a+** | **IFN-γ+** | **MIP-1β+** | **TNF-α+** |
| 127-33-1457-1080 | F | | UI | | 68.0 | 14.7 | 63.7 | 11.3 | 2.6 | 15.5 | 68.7 | 85.1 | 1.4 | 17.2 | 5.7 | 31.1 | 0.6 |
| 127-33-1782-1347 | F | | UI | | 70.9 | 11.2 | 73.0 | 16.7 | 2.3 | 6.0 | 76.7 | 64.8 | 1.5 | 13.1 | 3.0 | 34.4 | 1.3 |
| 127-33-1896-1440 | F | | UI | | 82.8 | 12.5 | 81.8 | 10.6 | 3.6 | 12.8 | 63.9 | 54.5 | 1.8 | 17.9 | 7.1 | 41.3 | 2.9 |
| 127-33-1854-1412 | F | | UI | | 72.9 | 11.6 | 68.4 | 19.1 | 3.2 | 37.5 | 47.3 | 73.4 | 7.5 | 22.0 | 3.6 | 41.9 | 3.1 |
| AS30-0018 | F | | VC+ | | 56.8 | 18.7 | 55.1 | 15.7 | 2.1 | 32.5 | 10.7 | 54.8 | 0.8 | 38.0 | 21.7 | 42.3 | 13.0 |
| FRESH  127-33-0397-268 | F | | VC+ | | 75.7 | 20.1 | 64.4 | 39.7 | 5.4 | 29.0 | 60.9 | 66.6 | 0.9 | 14.3 | 12.4 | 42.0 | 3.9 |
| SK-453 | M | | VC+ | | 30.4 | 20.4 | 83.3 | 19.2 | 2.2 | 22.4 | 18.9 | 40.8 | 0.7 | 2.9 | 4.3 | 37.0 | 3.3 |
| SK-235 | M | | VC+ | | 71.5 | 21.0 | 39.3 | 28.1 | 3.6 | 38.5 | 32.0 | 69.9 | 1.8 | 26.1 | 10.2 | 39.8 | 4.0 |
| 206-30-0011-0 | F | | VC+ | | 52.0 | 37.0 | 85.3 | 32.1 | 7.5 | 40.4 | 3.8 | 41.8 | 0.5 | 20.0 | 6.9 | 17.7 | 4.7 |
| 206-30-0020-0 | M | | VC+ | | 50.6 | 27.1 | 63.4 | 26.3 | 3.7 | 13.7 | 56.1 | 42.4 | 0.6 | 17.3 | 5.2 | 19.2 | 2.0 |
| 206-30-0012-0 | F | | VC+ | | 40.5 | 46.1 | 56.3 | 19.5 | 2.7 | 35.1 | 2.4 | 36.0 | 5.4 | 21.2 | 7.4 | 27.6 | 2.0 |
| 111-30-0005-0 | F | | VC+ | | 73.0 | 25.7 | 56.0 | 18.6 | 4.8 | 33.0 | 40.9 | 56.4 | 1.8 | 19.4 | 2.5 | 31.7 | 1.7 |
| 206-30-0007-0 | F | | VC+ | | 63.6 | 23.3 | 21.7 | 36.4 | 3.3 | 53.2 | 4.7 | 73.3 | 3.8 | 36.0 | 15.1 | 35.2 | 6.4 |
| SK-362 | F | | VC+ | | 85.4 | 17.5 | 55.1 | 30.9 | 8.0 | 43.6 | 35.1 | 60.9 | 6.7 | 26.7 | 9.2 | 29.8 | 1.5 |
| 111-30-0015-0 | F | | VC+ | | 42.9 | 19.9 | 62.4 | 28.2 | 3.0 | 27.9 | 54.5 | 66.4 | 2.3 | 8.2 | 2.0 | 17.8 | 1.0 |
| SK-317 | F | | VC- | | 33.6 | 47.0 | 45.2 | 14.3 | 7.0 | 35.2 | 53.5 | 32.1 | 0.5 | 21.0 | 17.7 | 41.4 | 8.7 |
| 206-30-0002 | F | | VC- | | 35.1 | 31.7 | 69.6 | 24.8 | 4.2 | 13.7 | 66.7 | 52.8 | 7.5 | 4.9 | 2.3 | 18.5 | 0.4 |
| 111-30-0041-0 | F | | VC- | | 55.7 | 21.8 | 58.1 | 42.6 | 4.3 | 21.3 | 61.4 | 51.7 | 2.0 | 1.6 | 0.0 | 11.6 | 0.1 |
| 206-30-0005-0 | F | | VC- | | 55.8 | 36.5 | 11.8 | 22.5 | 1.7 | 47.1 | 10.0 | 75.6 | 2.0 | 25.5 | 7.3 | 17.1 | 1.5 |
| 206-30-0004-0 | F | | VC- | | 54.1 | 33.8 | 58.7 | 32.6 | 6.6 | 15.1 | 7.3 | 32.7 | 1.8 | 14.7 | 4.2 | 36.4 | 0.6 |
| SK-275 | F | | VC- | | 96.7 | 36.9 | 63.6 | 21.3 | 20.4 | 26.5 | 58.8 | 56.8 | 1.1 | 28.2 | 12.2 | 18.4 | 2.6 |
| FRESH  127-33-0035-039 | F | | VC- | | 85.5 | 18.5 | 28.9 | 27.6 | 5.8 | 44.8 | 27.7 | 65.9 | 1.4 | 26.0 | 7.7 | 20.1 | 3.2 |
| 206-30-0024-0 | F | | VC- | | 37.2 | 77.0 | 54.9 | 28.4 | 12.2 | 9.8 | 38.5 | 44.4 | 1.9 | 0.7 | 0.4 | 11.2 | 0.2 |
| SK-209 | F | | VC- | | 79.5 | 34.7 | 43.6 | 12.8 | 17.4 | 56.1 | 43.6 | 64.3 | 4.3 | 0.3 | 0.4 | 3.7 | -1.4 |

^a^ UI, Healthy Uninfected controls; VC+, Viraemic controller with protective HLA-I alleles; VC-, Viraemic controllers without protective HLA-I alleles.
