# Supplementary material for: Differing natural killer cell, T cell and antibody profiles in antiretroviral-naive HIV-1 viraemic controllers with and without protective HLA alleles
Source: PLoS One. 2023 Jun 2;18(6):e0286507. doi: 10.1371/journal.pone.0286507 (PMC10237385; doi:10.1371/journal.pone.0286507)
Supplement: S5 Table — (DOCX) [file pone.0286507.s008.docx]

**S5 Table: Percentage of expression of different surface markers and intracellular cytokines within the CD3+ population.**

|  |  |  |  |  |  |  |  |  |
| --- | --- | --- | --- | --- | --- | --- | --- | --- |
| PID | **Sex** | **Role^a^** | **CD38+** | **CD57+** | **CD69+** | **HLA-DR+** | **PD-1+** | **HLA-DR+ CD38+** |
| 127-33-1457-1080 | F | UI | 38.0 | 19.1 | 4.5 | 6.0 | 19.1 | 0.7 |
| 127-33-1782-1347 | F | UI | 37.4 | 15.8 | 14.5 | 11.0 | 14.7 | 2.2 |
| 127-33-1896-1440 | F | UI | 56.7 | 13.8 | 5.8 | 5.9 | 19.0 | 1.0 |
| 127-33-1854-1412 | F | UI | 37.1 | 11.8 | 9.3 | 7.7 | 8.9 | 1.4 |
| AS30-0018 | F | VC+ | 18.3 | 23.4 | 16.5 | 11.4 | 25.6 | 1.5 |
| FRESH  127-33-0397-268 | F | VC+ | 47.1 | 17.8 | 29.1 | 21.5 | 17.2 | 9.1 |
| SK-453 | M | VC+ | 26.9 | 25.8 | 8.6 | 11.2 | 34.8 | 1.4 |
| SK-235 | M | VC+ | 31.5 | 17.0 | 15.2 | 12.7 | 17.0 | 2.2 |
| 206-30-0011-0 | F | VC+ | 28.1 | 12.2 | 29.6 | 16.1 | 14.0 | 1.8 |
| 206-30-0020-0 | M | VC+ | 6.8 | 49.4 | 19.5 | 19.5 | 18.3 | 1.1 |
| 206-30-0012-0 | F | VC+ | 19.6 | 9.2 | 24.7 | 7.7 | 24.1 | 1.5 |
| 111-30-0005-0 | F | VC+ | 33.0 | 13.3 | 19.8 | 9.5 | 24.1 | 2.0 |
| 206-30-0007-0 | F | VC+ | 16.0 | 26.7 | 15.4 | 24.5 | 13.1 | 2.1 |
| SK-362 | F | VC+ | 44.4 | 25.9 | 28.6 | 25.5 | 43.2 | 8.6 |
| 111-30-0015-0 | F | VC+ | 20.6 | 11.4 | 12.8 | 7.3 | 13.7 | 1.0 |
| SK-317 | F | VC- | 25.0 | 40.5 | 12.0 | 24.4 | 23.7 | 3.2 |
| 206-30-0002 | F | VC- | 18.4 | 19.1 | 17.8 | 19.6 | 23.1 | 1.8 |
| 111-30-0041-0 | F | VC- | 20.7 | 32.6 | 17.8 | 15.3 | 19.4 | 2.2 |
| 206-30-0005-0 | F | VC- | 22.2 | 32.1 | 15.9 | 14.7 | 17.3 | 2.1 |
| 206-30-0004-0 | F | VC- | 28.3 | 26.3 | 35.2 | 28.9 | 20.4 | 5.8 |
| SK-275 | F | VC- | 42.9 | 23.1 | 20.6 | 15.7 | 19.8 | 5.5 |
| FRESH  127-33-0035-039 | F | VC- | 27.1 | 35.3 | 23.8 | 19.4 | 26.9 | 3.7 |
| 206-30-0024-0 | F | VC- | 24.7 | 16.3 | 17.2 | 24.6 | 22.5 | 2.6 |
| SK-209 | F | VC- | 32.0 | 23.4 | 11.9 | 33.2 | 17.6 | 9.5 |

^a^ UI, Healthy Uninfected controls; VC+, Viraemic controller with protective HLA-I alleles; VC-, Viraemic controllers without protective HLA-I alleles
